# Molecular mechanism of lateral bud differentiation of *Pinus massoniana* based on high-throughput sequencing

**DOI:** 10.1038/s41598-021-87787-7

**Published:** 2021-04-27

**Authors:** Hu Chen, Jianhui Tan, Xingxing Liang, Shengsen Tang, Jie Jia, Zhangqi Yang

**Affiliations:** 1 Guangxi Forestry Research Institute of Science, Nanning, 530002 People’s Republic of China; 2 Guangxi Key Laboratory of Superior Timber Trees Resource Cultivation, Nanning, 530002 People’s Republic of China; 3 Engineering Research Center of Masson Pine of State Forestry Administration, Nanning, 530002 People’s Republic of China; 4 Engineering Research Center of Masson Pine of Guangxi, Nanning, 530002 People’s Republic of China

**Keywords:** Plant biotechnology, Plant development, Plant genetics, Plant molecular biology, Plant signalling

## Abstract

Knot-free timber cultivation is an important goal of forest breeding, and lateral shoots affect yield and stem shape of tree. The purpose of this study was to analyze the molecular mechanism of lateral bud development by removing the apical dominance of *Pinus massoniana* young seedlings through transcriptome sequencing and identify key genes involved in lateral bud development. We analyzed hormone contents and transcriptome data for removal of apical dominant of lateral buds as well as apical and lateral buds of normal development ones. Data were analyzed using an comprehensive approach of pathway- and gene-set enrichment analysis, Mapman visualization tool, and gene expression analysis. Our results showed that the contents of auxin (IAA), Zea and strigolactone (SL) in lateral buds significantly increased after removal of apical dominance, while abscisic acid (ABA) decreased. Gibberellin (GA) metabolism, cytokinin (CK), jasmonic acid, zeatin pathway-related genes positively regulated lateral bud development, ABA metabolism-related genes basically negatively regulated lateral bud differentiation, auxin, ethylene, SLs were positive and negative regulation, while only A small number of genes of SA and BRASSINOSTEROID, such as *TGA* and *TCH4*, were involved in lateral bud development. In addition, it was speculated that transcription factors such as WRKY, TCP, MYB, HSP, AuxIAA, and AP2 played important roles in the development of lateral buds. In summary, our results provided a better understanding of lateral bud differentiation and lateral shoot formation of *P. massoniana* from transcriptome level. It provided a basis for molecular characteristics of side branch formation of other timber forests, and contributed to knot-free breeding of forest trees.

## Introduction

Branching, which is a common phenomenon, is characterized by a high level of plasticity to plant morphology. The number of branches determine the increase of nutrient organs, resulting in increase of plant biomass and plasticity in branching helps plants strengthen response to environmental changes^1,2^. Many factors affect formation of shoots, including genetics, hormones, development, light intensity, light characteristics and soil nutrients, besides, hormones play important roles in the process of branch formation^1,3^. Numerous studies have also shown that endogenous hormones such as auxin (IAA), cytokinins (CKs) and strigolactones (SLs) regulated branching structure and shoot development of plants with a series of complex and specific signal transduction pathways^4–6^.

IAA was found to be the first plant hormone involved in the regulation of shoot growth^7^. IAA is synthesized mostly in buds and young leaves, and causes an indirect inhibitory effect on development of lateral shoot^2,8,9^. In polar auxin transport, AUX/LAX proteins are needed to transport auxin into the cell, while PIN and PGP proteins are required to transport auxin out of the cell^10^. In *Arabidopsis*, IAA negatively regulates the synthesis of CKs via AXR1-mediated signaling^11^. SLs are new hormones that have unique roles in lateral bud outgrowth^12,13^. C*AROTENOID CLEAVAGE DIOXYGENASE (CCD7*), *CCD8*, *D27*, *MAX1 (Cytochrome P450)* play essential roles in SLs synthesis^5,12,14,15^. SL signaling is activated by transcription factors, such as *D14*, *MORE AXILLARY GROWTH 2 (MAX2)*, TCP transcript factors. CKs have direct effects on axillary bud outgrowth^16,17^. GA and ABA affect lateral shoot branching and regulation^18,19^. However, complete regulatory network is not clear^20^.

In addition, recently researches found that some transcription factors were involved in development of collateral. For example, AP2/ERF, WRKY, MYB, and TCP transcript factors were found to play important roles in the differentiation and elongation of lateral shoot^21–24^.

Woody plants, especially afforestation timber tree species, have different research purposes on lateral shoot development. Crops and ornamental plants with multiple branches are necessary to get high yield and beautiful appearance. Whereas the timer tree species are expected to have few or zero lateral shoot in order to cultivate large diameter and knot-free timber, increase output and utilization of timber, reduce processing cost, and enhance the appearance of solid wood products. Cultivation and physiology on lateral bud development have been studied by many researches^25^, Lei and Sumida^26^ studied the changes in branch structure of spruce under different light conditions. Researches on lateral bud development of polar in molecular side has been studied. The role of *DWARF14*, *BRANCHED1* and other genes^27,28^ in SL signaling regulation were determined. Wang et al.^29^ preliminarily revealed the relationship between activity of lateral buds and hormones in hybrids of *Populus euramericana* and poplar using transcriptomics. At present, the research on the regulation mechanism of collateral development of woody plants has just begun, and the theoretical research on the mechanism of hormonal regulation of lateral bud differentiation will become the key direction.

*P. massoniana* is a typical coniferous native tree species in southern China with strong adaptability, fast growth rate, which provides 16% forest growing stock in southern China and rosin production accounts for more than half of the world's turpentine^30,31^. *P. massoniana* has more thick lateral shoot than *Pinus elliottii*, *Pinus taeda* and *Larix potaninii*, which is not easy to pruning and unfavorable for knot-free timber cultivation. Many researches about density regulation and pruning have been carried out, however, there have not been any reports on the selection of superior varieties of knot-free timber. Therefore, the analysis of development mechanism of lateral shoot is the best way to improve quality of *P. massoniana* wood.

In our study, we observe the process of lateral bud differentiation by microtechnique, and analyzed hormone changes during lateral bud development as well as transcriptomes of terminal bud and lateral bud after decapitation and in normal growth plants by high-throughput sequencing technology (control). We identified differentially expressed genes (DEGs) by reference and de novo assembly methods. Functional enrichment analysis of these DEGs indicates that lateral bud differentiation is affected by hormones and regulated by related genes, which may be the putative regulatory center of lateral bud differentiation. Our results provide important genetic resources and theoretical basis for differentiation mechanism of *P. massoniana*, and provide a theoretical basis for knot-free timber cultivation.

## Result

### Effect of removal of apex on plant morphology

**Figure 1:**
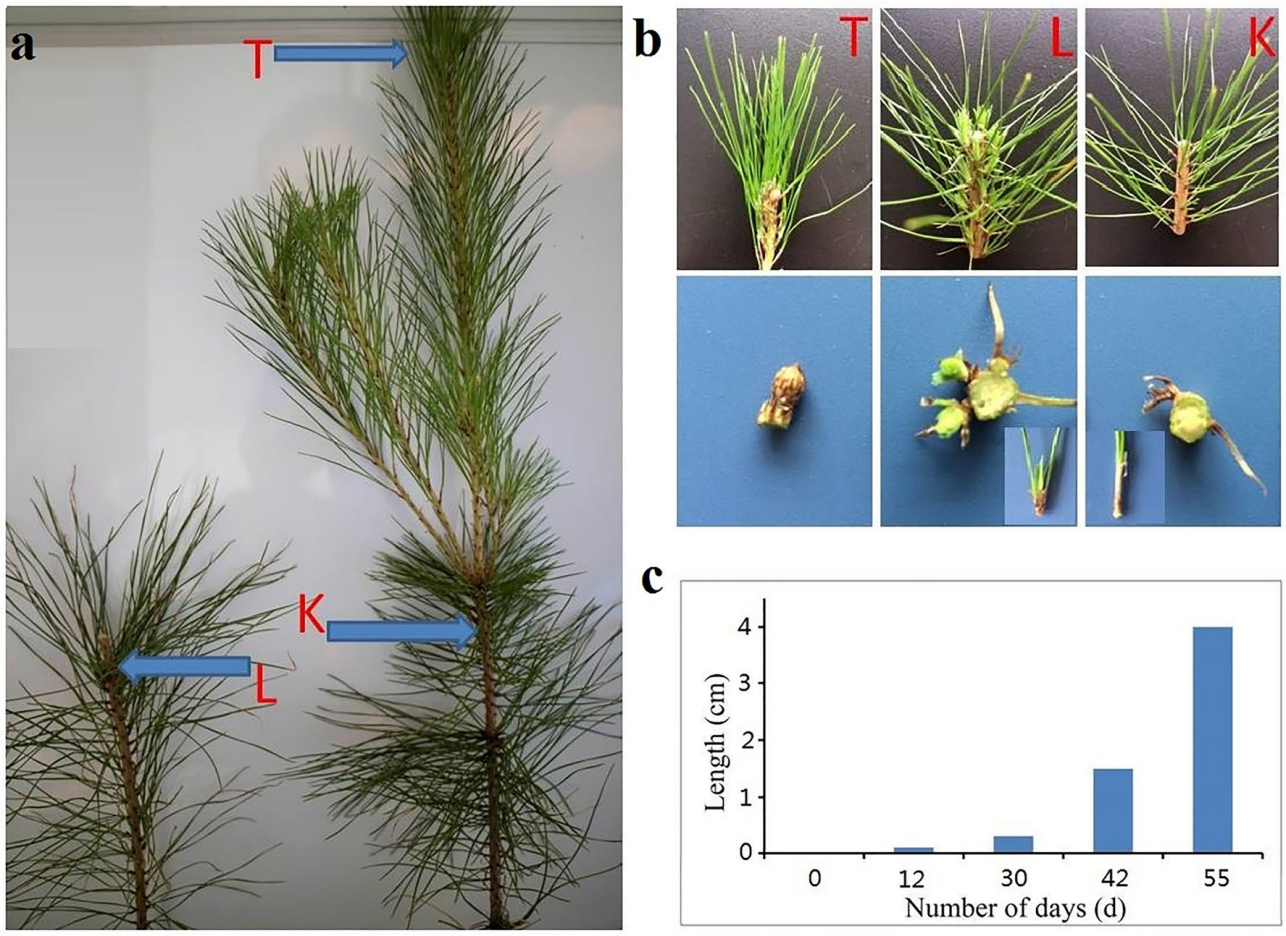
Effect of decapitation on phenotype of plants. (**a**) Decapitation was carried out in mature needle leaves with apex shoots and kryptoblasts as control groups. (**b**) Lateral buds subjected to paraffin section and kryptoblasts with xylem, epidermis of samples which analyzed by RNA-seq; (**c**) growth situation of lateral buds after decapitation until 55 d.

The removal of apex results in plant height decrease from 55–60 cm to 35–40 cm (Fig. 1a). There were a great number of lateral bud outgrowth after 8–10 days, which were coming from a cluster of needle leaves (Fig. 1b). After removal of apical dominance, lateral buds grew rapidly with the length of 0.3 cm and 4 cm in12th and 55th day, respective.

### Morphological feature of buds

**Figure 2:**
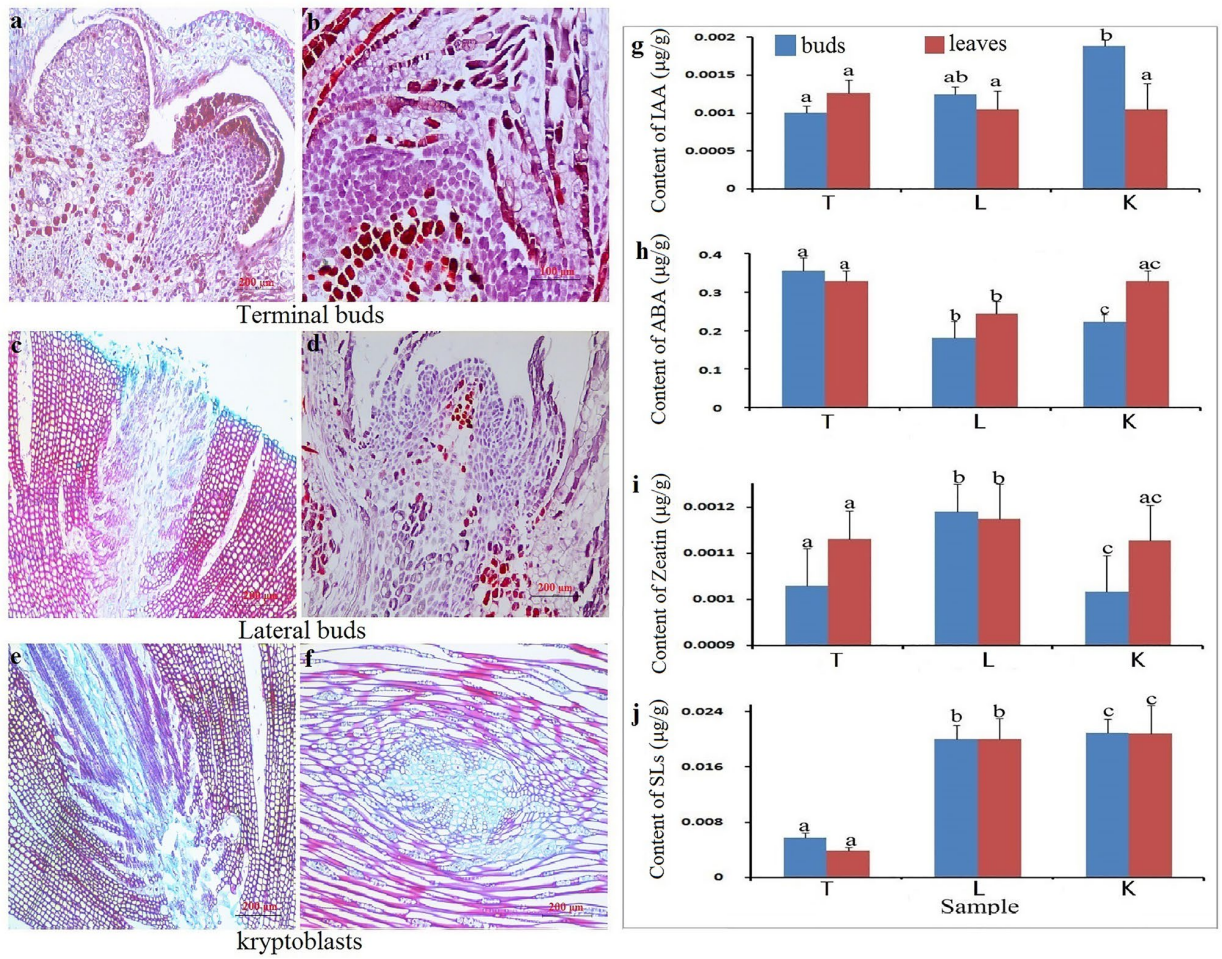
Microscopic observation of lateral bud differentiation and determination of hormone contents. (**a**) Paraffin section observation of apex buds, lateral buds and kryptoblasts using microscope (100 ×). (**b**) Analysis of IAA, ABA, Zea, SLs using UPLC-MS–MS, blue columns represented hormone contents of apex buds, lateral buds and kryptoblasts and red columns represented hormone content of needle leaves. Every sample had three replication. Different letters indicated significant differences (ANOVA, LSD, *P* < 0.05).

The bud tip of *P. massoniana* included meristematic zone, elongation zone and maturarion zone (Fig. 2a). In ball-shape meristematic zone, the promeristem was composed of tunica cells and corpus cells. Tunica cells have longitudinal division to enlarge superficial area while irregular corpus cells undergo longitudinal and transverse divisions, amplifying volume of stem tip (Fig. 2b). Vascular bundle was composed of primary phloem, fascicular cambium and primary xylem, and meristematic cell in fascicular cambium had strong activity, and grew out of periderm along direction of phloem rays and became lateral buds (Fig. 2c,d). As can be seen from Fig. 2e and f, axillary buds originated from parenchyma cells of vascular cambium. The flat and rectangular-shape fusiform cells, distributed in ascicular cambium, were the main bodies of vascular cambium. Axillary buds have formatted during normal growth and development, but apical dominance has not been stimulated.

### Contents of hormones in different parts

The order of IAA content was apical buds, lateral buds and kryptoblasts. The content of IAA was significantly different between apical buds and kryptoblasts (*P* < 0.05). The content of IAA was also the highest in needle leaves around apical buds, but there were no significant differences compared to other materials (Fig. 2b). The highest and the lowest amount of ABA was in apical buds and lateral buds, respectively, and there were significant differences among other ones (P < 0.05). The needle leaves also showed the same phenomenon (Fig. 2b). Content of Zea was the highest in the lateral buds and leaves around lateral buds, and there is significant difference compared with other materials (Fig. 2b). The lowest content and the highest content of SLs were in shoot apex and leaves around shoot apex, axillary buds, respectively. Although SL contents in lateral buds were closed to those of axillary buds, there were significant differences (P ≤ 0.05.

### Transcriptome data assembling and annotation

We sequenced a total of 9 c-DNA libraries from 3 samples (in duplicates). We obtained a total of 232,736,473 high-quality reads containing 69,512,197,035 bases with GC contents of 45–48% (Supplementary Table S1).

All data were assembled by the Trinity software, resulting in 756, 676 Transcripts and 604, 122 unigenes with N50 of 801 and 395, respectively. The average length of unigene was more than 200 bp, and the length of 200–500 bp unigenes occupied 85.4% (Fig. S1, Table S2). 355, 314 unigenes were higher similar to known proteins from seven above-mentioned protein databases (Table S3). To further identify the interactions of all the annotated unigenes, we carried out GO functional enrichment and KEGG pathway analysis. A total of 174, 942 were assign to the three main GO categories (Fig. S2). Also, and total of 1219, 4910, 4628 from L v T, L v K and K v T were assign to KEGG pathways (Fig. S2, Table S4).

### Differential expression analysis and differentially expressed genes (DEGs) screening

**Figure 3:**
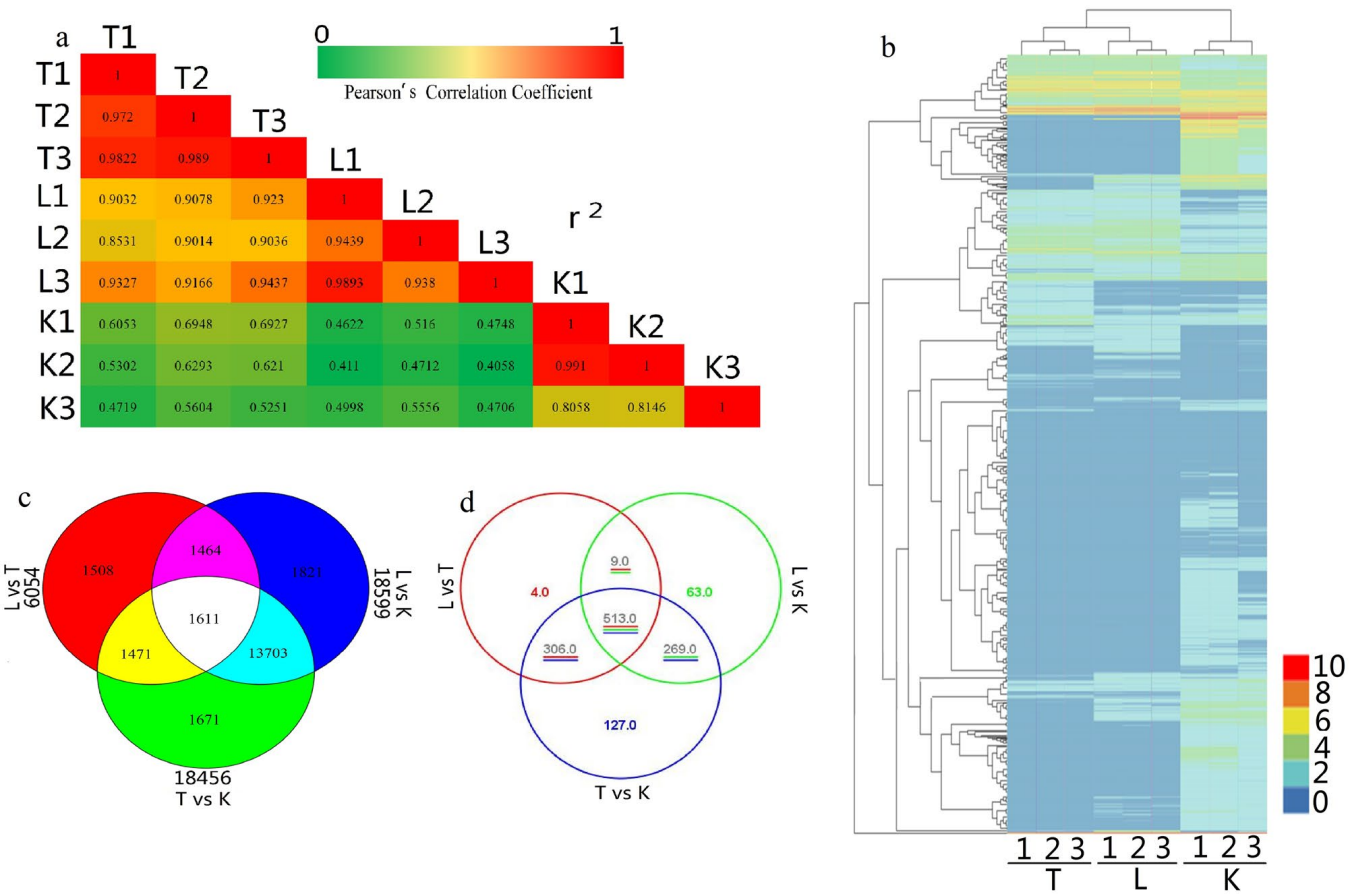
Expression analysis of DEGs. (**a**) In heat map, different columns represented different samples, different rows represented different genes, and colors represented the value of FPKM. (**b**) Veen clustering of All_DEGs. (**c**) In order to re-annotate All_DEGs sequence in Mapman software, Mapman can be annotated and meets the threshold, which will only appear if the gene-related expression that can be annotated to the specific pathway meets the criteria. It required two sets of samples to be greater than 2 or less than -2 at the same time. (**d**) Numbers of up-regulation and down-regulation genes in different samples. (**e**) Represented biological repeat correlation statistics. Pearson's Correlation Coefficient (r) was used as an indicator of biological repetitive correlation. The closer is r^2^ to 1, the stronger the correlation between the two replicates^58^.

Compared with T and K samples, we identified 3322 unigenes and 3826 unigenes showing up-regulation as well as 1574 unigenes and 13,146 unigenes indicating down-regulation in L sample (Fig. 3d), respectively. Pearson’s Correlation Coefficient (r) of three replications in L, T, K samples were 0.938, 0.972, 0.8058, respectively, indicating good replication (Figs. 3, S3). Meanwhile, we found that the r between L and T was more than 0.8531, which showed that expression of these two samples was similar (Figs. 3a,e, S3).

In DEGs, compared with T and K samples, we identified 3934 and 4395 DEGs showing up-regulation as well as 2120 and 14,204 DEGs indicating down-regulation in L sample, respectively. Compared K and T, we examined 16,126 DEGs showing up-regulation and 2330 DEGs showing down-regulation (Fig. 3b, Table S5). Gene ontology analysis was performed by mapping each unigene to the GO database. 12.37% unigenes were annotated in Mapman and found to be associated with 34 metabolism pathways (Figs. 3c, Fig. S4, Table S6). 90% DEGs were found to be similar to known protein from Nr database to provide basis for metabolism analysis (Fig. S2, Table S7).

### Differential expression of hormone signaling pathway related genes

**Figure 4:**
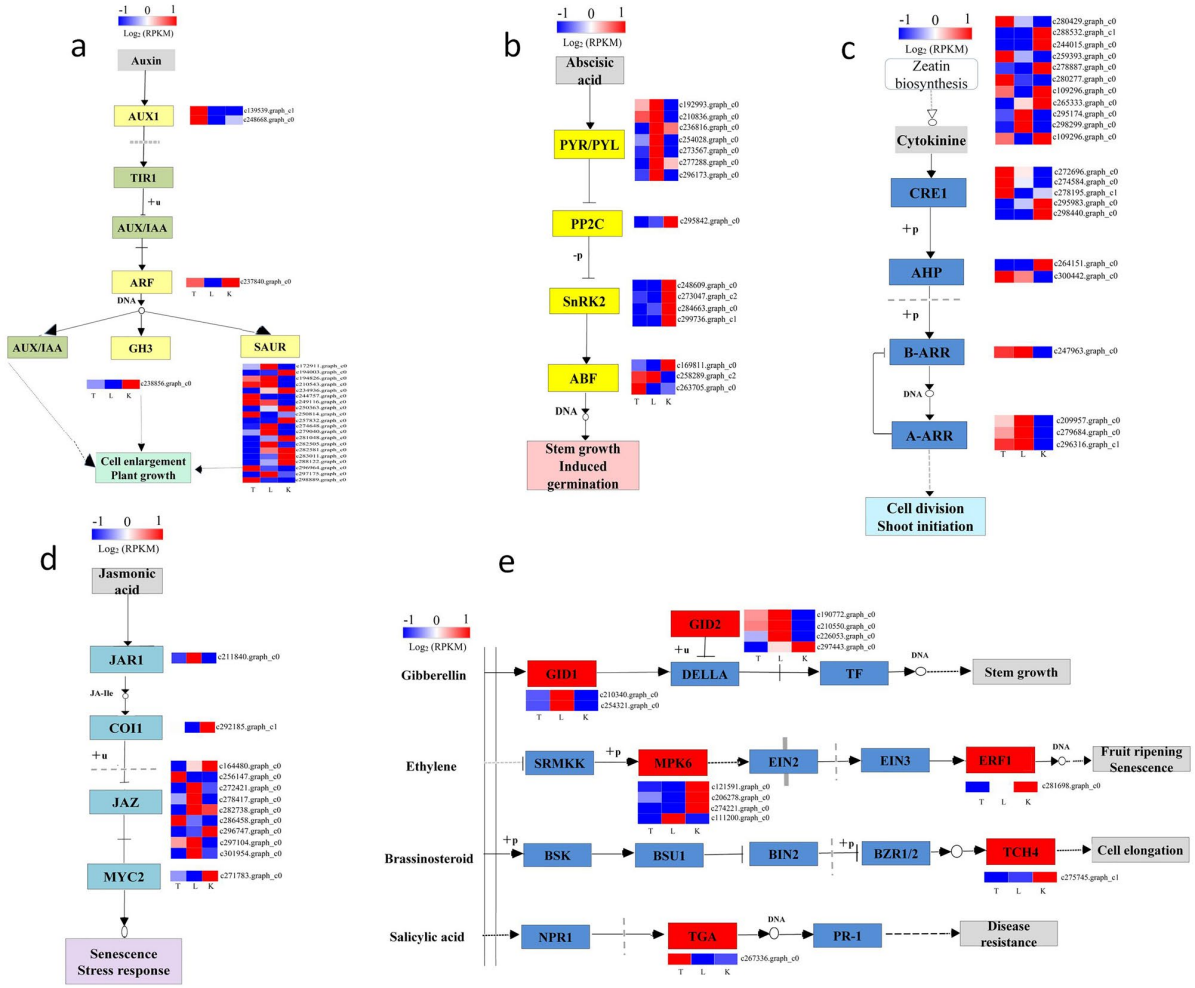
Analysis of hormonal metabolic pathways of differentially expressed genes (DEGs) involved in lateral bud differentiation. (**a**) Auxin synthesis pathway; (**b**) Abscisic acid synthesis pathway; (**c**) Cytokinin synthesis pathway; (**d**) Jasmonic acid synthesis pathway; (**d**) Metabolic pathway of gibberellin, ethylene, brassinosteroid, and salicylic acid. The FPKM ratio of unigene expression was represented on a logarithmic scale for each treatment (T, L, K). Red indicated that a gene was up-regulated at that stage, whereas green indicated down-regulated expression. The heat map was conducted by morpheus (https://software.broadinstitute.org/morpheus/).

Since different hormone signaling pathways have antagonistic effects on lateral bud differentiation, it is important to study the response of plant hormones. We found that genes of signaling pathways of IAA, ABA, cytomin, jasmonic acid, GA, ethylene, brassin steroids and salicylic acid (SA) were expressed. Auxin (*Aux1*), Auxin response factor (*ARF*), GH3 auxin-responsive promoter (*GH3*) of IAA negatively regulated lateral bud development except for *SAURs* with both positive and negative regulation (Fig. 4a, Table S8). ABA receptor (*PYR/PYL*), Protein phosphatase 2C (*PP2C*), *SNRK2*, *ABF* from ABA (Fig. 4b, Table S8), *CKX/CYP735A/CISZOG* from zeitin, *GRE1*, *AHP*, *B-ARR*, *A-ARR* from cytomin, *JAR1*, *COI1*, *JAZ*, *MYC2* from jasmonic acid (Fig. 4c, Table S8), GA receptor GID (*GID1*) and *GID2* from GA, ethylene-responsive transcription factor (*ERF1*), TGA from SA, *TCH4* from brassin steroid were up-regulated differentiation of lateral buds, while *MPK6* from ethylene were down-regulated (Fig. 4d,e, Table S8). Moreover, alpha/beta hydrolase DWARF (ABHD17B) and esterase D14L (D14/KAI2/DAD2) from SLs were up-regulated, while carotenoid cleavage dioxygenases (*CCD*), putative 9-cis-epoxycarotenoid dioxygenase9 (NCED), NCED1 were down-regulated.

### Differential expression of transcription factor-related genes

**Figure 5:**
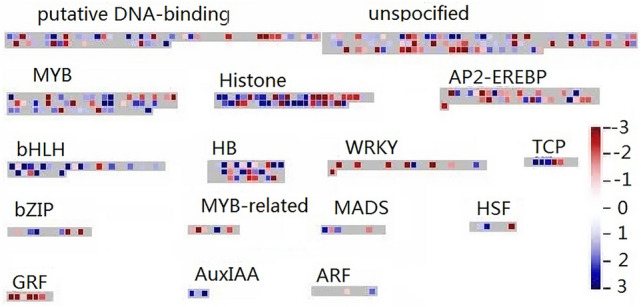
Differential expression of transcription factor-related genes. The pathway was generated using MapMAN software (Version 3. 6.0RC1). Totally 901 genes were involved in different transcription factor pathway. Each square means a gene. Blue color means up-regulation and red color means down-regulation. Data was from differential expression of L v K.

We used Mapman software to understand the involvement of transcription factors during the development of lateral buds after top treatment. The results showed that 901 genes from 55 transcription factors were involved in the lateral bud development (Figs. 5, S5, Table S9). *WRKY49*, *WRKY1*, *TCP1*, *TCP4*, *TCP20* from L v K sample, *WRKY31*, *WRKY41*, *WRKY48*, *WRKY1* from L v T sample, *MYB13*, *MYB30*, *MYB308*, *MYB4* were up-regulation genes. While *WRKY50* and *WRKY51* from L v K sample, NAC family TFs, *MYB3*, *MYB9*, *MYB11*, *MYB2* were almost down-regulation genes.

*DAL21* (MADS family) showed up-regulated expression, and all others were down-regulated. HSP family TFs all showed up-regulated expression, which showed positive regulation of lateral bud development. Only Glycine-rich RNA-binding protein 2 (*GRP2*, GRF family) showed negative regulation of lateral bud development. The AuxIAA family TFs was expressed as a positive regulation of lateral bud development, and ARF2 and ARF19 (Auxin response factor) (ARF family) appeared as negative regulatory genes. The ethylene-responsive transcription factor LEP *(ERE-LEP)* was a positive regulatory gene in AP2-EREBP family TFs, and *ERE3*, *ERE23*, *ERE38*, and *ERE12* negatively regulated lateral bud development. Overall, our study showed that a large number of transcription factors were involved in development of lateral buds, some families were all positively regulated, some families had positive and negative regulatory genes, and key genes in several families TFs have been identified.

### Differential expression of genes related to secondary metabolism

**Figure 6:**
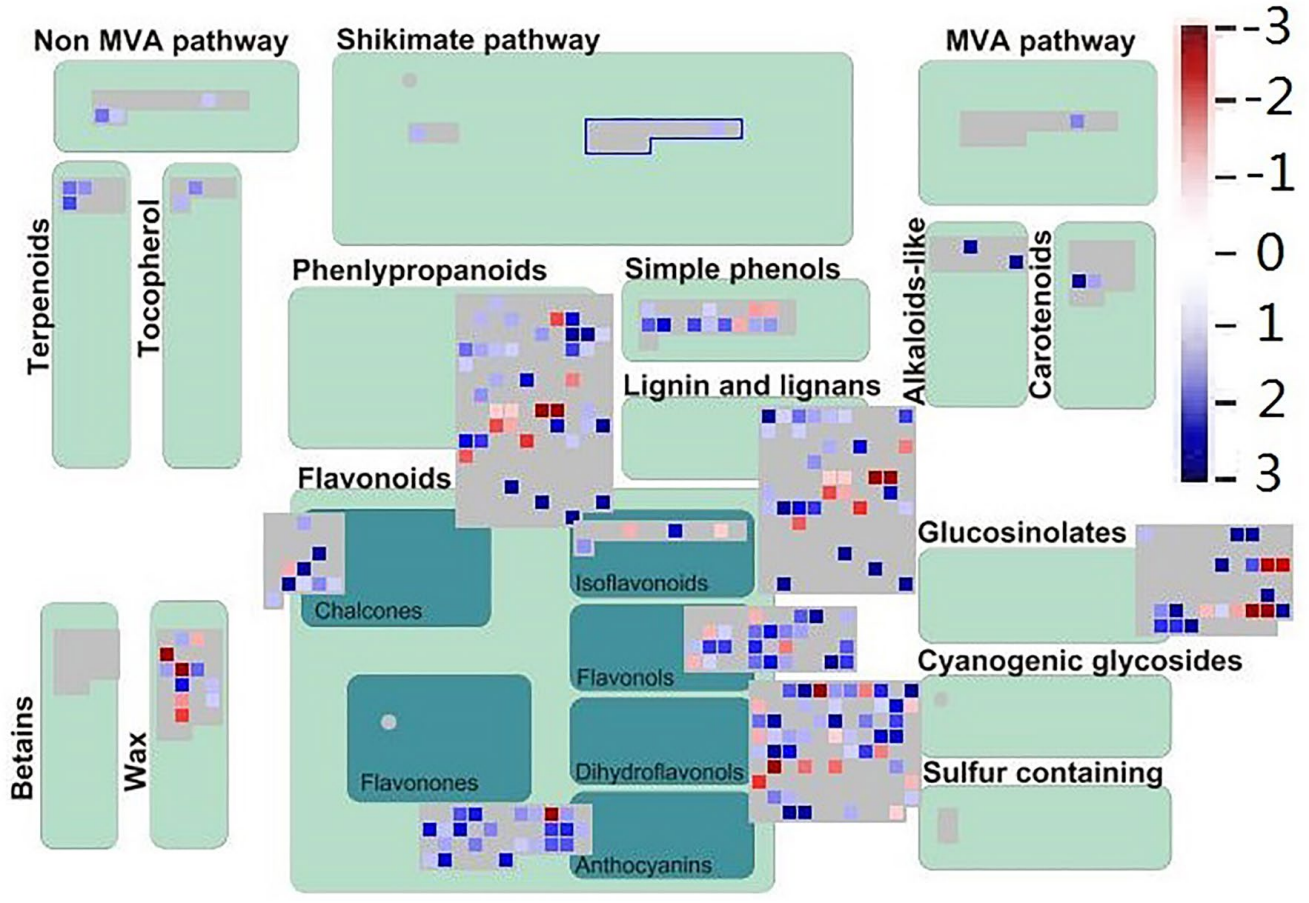
Differential expression of genes related to secondary metabolism. The pathway was generated using MapMAN software (Version 3. 6.0RC1). Totally 701 genes were involved in different pathway. Each square means a gene. Blue color means up-regulation and red color means down-regulation。Data was from differential expression of L v K.

We used Mapman software to understand the involvement of transcription factors in the development of lateral buds after cutting, and the results showed that 701 genes involved in 19 secondary metabolic pathways including carotenoids, chalcones, lignin and lignans, MVA pathway, Non MVA pathway, and terpenoids were involved lateral bud development (Figs. 6, S6, Table S10). In L v D sample, we found that carotenoids was up regulation; while in L v K sample, except for cytochrome P450 family monooxygenase, other genes were down regulation. Some genes negatively regulated, including acetyl-CoA C-acetyltransferase, acetyl-CoA synthetase-like protein, alcohol dehydrogenase, caffeic acid ortho-methyltransferase, cinnamoyl-CoA reductase, GroES-like protein, NADP-dependent alcohol dehydrogenase from lignin and lignans pathway, e acetyl-CoA C-acetyltransferas from MVA pathway, other ones showed positively regulated, such as hydroxycinnamoyl CoA shikimate, phenylalanoyl CoA ligase, geranylgeranyl transferase and 1-seoxy-d-xylulose 5-phosphate synthase genes from non MVA pathway, isopimaradiene synthase, limonene synthase, longifolene synthase and other genes from terpenoids metabolic pathway.

### qRT-PCR

**Figure 7:**
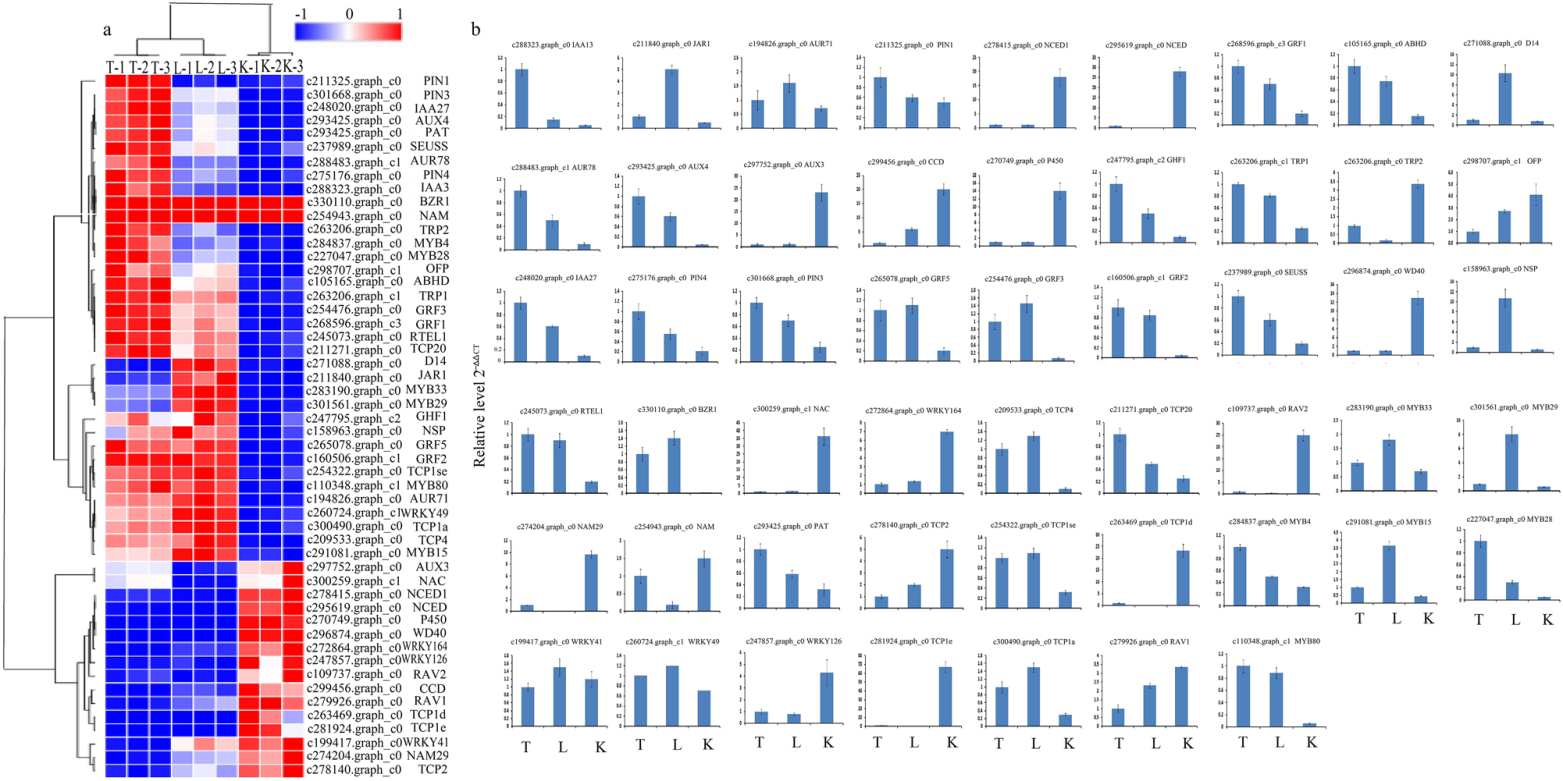
Real-time PCR validations of 52 candidate DEGs in different material. (**a**) Heat map of 52_DEGs. Different column represented disserent samples and different rows represented various genes. (**b**) qRT-PCR of 52_DEGs. The Y-axis represents the relative expression, and the X-axis depicts the different insect resistance material. The standard error of the mean for three biological replicates is represented by the error bars.

To test the reliability of RNA-Seq data, we selected 53 DEGs to be examined by real-time quantitative (qRT-PCR). These genes included genes related to AUXIN resistant, Phytohormone auxin, carotenoid cleavage dioxygenases, More axillary growth, alpha/beta hydrolase, Sigaling repressor, Decreased apical dominance, which played key roles in lateral bud development, as well as genes from WRKY, TCP, MYB, RAV. The qRT-PCR results were consistent with RNA-Seq (Fig. 7), indicating that the RNA-Seq sequencing results were accurate and effective.

## Discussion

At present, there are few researches on the growth, development and stress response mechanisms of *P. massoniana*^32,33^. Development of lateral shoot is an important factor affecting the growth, product and quality of *P. massoniana*, but studies on response mechanism and molecular response mechanism are rarely reported. The sequencing technology provides us a direct insight into the genes response and allowi us to systematically, rapidly and comprehensively elucidate the molecular mechanisms for development of lateral shoot. The study used high-throughput sequencing technology to obtain a total of 756, 676 Transcript and 604, 122 Unigene. In addition, through the comparative analysis of materials, a large number of DEGs related to the lateral bud differentiation of *P. massoniana* were identified. The annotated DEGs covered plant growth and development, signal transduction, transcriptional genes, and secondary biomass biological processes. It provides basic for the molecular mechanism of lateral shoot differentiation of *P. massoniana*.

The growth regulation of lateral buds is complex, and plants need to respond to multiple exogenous and endogenous signals simultaneously^34^. The growth of lateral shoot is regulated by a variety of endogenous hormones, including auxin and SLs, which inhibit the growth of lateral buds, and CKs, which promote the growth of lateral buds. The studies of a large number of model plants aim to explore interaction and effects of three hormones in axillary buds. The interaction between these hormones forms a balanced network to regulate lateral shoot development^2,35^.

After decapitation of apex, the reduce of auxin synthesis results in decrease in auxin content that transported downward, thereby inducing lateral bud outgrowth. In our study, the content of IAA in K sample was high, and the IAA content in the L sample also increased after removal of apical dominance, which indicated that inhibition of the lateral bud was removed after topping^9^. SLs are considered to be new hormones that directly regulate plant branching^12,14^. Our study found that zea also played a role in lateral bud development, and Zea was a prerequisite for CK synthesis^36^.

The interaction between auxin and SLs was found to be the most important mechanism in plant lateral shootl differentiation. Auxin polar transport (PATS) and the second messenger model were accepted to explain regulation^37,38^.

The discovery of multiple mutants accelerated the study of auxin-SLs pathway-associated genes and overall regulatory networks^2^. The auxin signal mainly relies on PIN, PGP, AUX1/LAX for transport and conduction, and regulates the synthesis process of SLs by regulating AUXIN RESISTANCE PROTEIN 1–AUXIN SIGNALLING F-BOX PROTEIN (*AXR1-AFB*) gene. CAROTENOID CLEAVAGE DIOXYGENASE (carotenoid cleavage double oxygenase 7, *CCD7*), *CCD8* (carotenoid cleavage double oxygenase 8), *D27* (a ferritin), *MAX1* (Cytochrome P450) were involved in the biosynthesis process of SLs. Carotenoids synthesized active SL 5-deaza-dual-gold alcohol, and then synthesized a variety of sole gold. Lactones by a series of biochemical processes to synthesize the^5,12,15^. Signaling transduction genes of SLs included *D14* (encoding α-β folding hydrolase), *MORE AXILLARY GROWTH 2* (*MAX2*, encoding SCF complex F-box protein, also known as Leu-rich repeat F-box protein), TCP transcription factor, etc.

These literatures verified our research data. In our study, a large number of signaling pathway genes were activated and exhibited different expression patterns, indicating that they had various functions in regulation of collateral differentiation. Among them, D alpha/beta hydrolase (*DWARF*)^39^, D14^40^, carotenoid cleavage dioxygenases (*CCD*)^24^, MORE AXILLARY GROWTH (*MAX*)^41^ of SL signaling pathway have been shown to be importance in collateral differentiation.

In addition, we also found that some hormone signaling pathway genes except for auxin, SL, and ABA were involved in the lateral shooting of *P. massoniana*, such as *GID* from GA metabolism, *ERF1* from ethylene pathway, *TGA* from SA and *TCH4* frome Brassinosteroid pathway. Although IAA, GA and ABA were involved in collateral germination and regulation^42,43^, regulatory mechanism was still unclear.

Transcription factors were another important regulatory genes that regulated gene expression. A series of physiological, biochemical, metabolic and defense mechanisms have been formed, which played important roles in plant growth and development and in adapting to stresses. There were 75 WRKY transcription factors regulating plant growth, senescence, hormonal signaling pathways in *Arabidopsis*, and these genes responsed to biotic and abiotic stresses^44^. Although we have a comprehensive understanding of hormone involvement in lateral bud differentiation, little is known about the role of transcription factors in collateral differentiation^2^.

Studies have shown that *WRKY71* regulates EXCESSIVE BRANCHES1 (*EXB1*) gene expression through auxin pathway and plays an important role in lateral bud differentiation, and also positively regulates lateral bud differentiation through H_2_O_2_, ABA signaling pathway in *Arabidopsis*. *WRKY71* increased the number of branches through up-regulated expression of *RAX2* and IAA efflux-related *PINI2* and confirmed the direct interaction with *RAX2*^22,23,45^. Studies have confirmed that *WRKY70* is an important member of salicylic acid and jasmonic acid-regulated defense signals. *WRKY70* was activated by salicylic acid and repressed by JA, and regulates SA and JA to participate in defense^26^. At the same time, many genes play negative regulatory roles in the defense process^46^, and *WRKY70* negatively regulated growth of lateral roots and root hairs in root development^47^. Our study showed that not all genes in the WRKY transcription factor family are up-regulated, such as *WRKY50*, *WRKY51*, and confirmed that *WRKY1* gene regulated insect-resistant defense of *P. massoniana* (data not shown) through jasmonic acid, GA and other hormonal signaling pathways. We hypothesized that WRKY transcription factors may also participate directly or indirectly in lateral bud development through one or several hormonal signaling pathways.

The BRANCHED1 (*BRC1*) from TCP family played an important role in Arabidopsis lateral shoot differentiation^48^, and PsBRC1 regulates collateral differentiation of pears via the SL and CK pathways^49^. Our study showed that the TCP transcription factor family gene showed up-regulated expression during lateral bud differentiation, indicating that TCP promotes collateral development, but whether it is regulated by SL or CK pathway remains to be further studied.

The tomatoTrifoliate (*Tf*) gene from tomato MYB transcription factor family plays an important role in the development of axillary buds, which was similar to LATERAL ORGAN FUSION1 (*LOF1*) and *LOF2* genes in *Arabidopsis*^50,51^. ERF BUD ENHANCER (*EBE*) gene from AP2/ERF transcription factor directly regulates the differentiation of *Arabidopsis collaterals*^52^, CmERF053 plays an important role in lateral shoot differentiation of chrysanthemum^21^. Loss of genes such as TB1, TCP18, TCP12, and BRC2 leads to an increase in collaterals^2,53^. We initially identified 55 transcription factors involved in the lateral bud differentiation of *P. massoniana*, the further verification of synergistic reaction in the lateral bud differentiation of *P. massoniana* is needed.

## Material and method

### Plant materials and experimental design

The fast-growing (average annual growth of 1.6 m, DBH of 2 cm) full-sib family of *P. massoniana* M1315 (the father is Gui GC305, the mother is Gui GC1502) seeds were provided by Guangxi Forestry Research Institute, Nanning, Guangxi. Cultivation, collection and use of these seeds were approved by the Academic Committee of Guangxi Forestry Research Institute and in full accordance with “*Seed zones of Chinese forest tree-Seed zones of Pinus Massoniana Lamb*.” (GB 8822.6-1988) of National Forestry and Grassland Administration. Seeds were harvested in November and planted with light-matrix non-woven fabrics technique in the nursery garden of Guangxi Forestry Research Institute in December, 2017. After breeding under outdoor condition for five months, seedlings were placed in an artificial climate chamber for cultivation (light/dark: 8/12 h, light intensity 1200 lx, air humidity 75%) with consistent management as previous one. After 15 days, we excised the shoot tip from shoot axis of six-m-old seedlings to form lateral buds (L), while terminal buds (T) from normal seedlings and kryptoblasts (K) on stems were chosen as control groups. Lateral bud samples were removed when the length was 0.05–0.1 cm (Fig. 1). Three biological replicates were used for each sample, and 10 seedlings were treated per sample. Samples for transcript analysis and fluorescence quantitative expression analysis were stored in liquid nitrogen. Microscopic observation samples were stored in FAA fixative (38% formaldehyde 5 mL, glacial acetic acid 5 mL, 70% alcohol 90 mL). Fresh samples were used for hormone analysis.

### Micro-observation

The fresh tissues were fixed in Formalin fix liquid–acetic acid–ethanol (FAA) for more than 24 h. The tissues were soften by acid solution, dehydrated by graded alcohol series with dehydrator, soaked wax, and placed them in embedding machine. After trimming paraffin blocks into appropriate size, we cut 4 ~ 10 μm sections from each sample. The paraffin ribbons were placed in water bath at 50 °C, flatterned and mounted onto slides, incubated in 60 °C and stored at room temperature. SafraninO-fast green was used for staining. Finally, examination was carried out by microscope (LEICA, DM3000, Germany), image acquisition was performed by software Leica Application Suite V4 and analyzed by image analysis system DMC4500+. All of the tools are from LEICA company, Germany.

### Measurement of hormone

0.1 g fresh leaves were collected to determined contents of IAA, zeatin and ABA, while 0.5 g fresh leaves were used to analyze SLs. All the hormones were determined by high-performance liquid chromatography (HPLC). Briefly, 2μL volume of filtrate was injected into the Agilent 6460 Triple Quadrupole LC/MS (America) and analyzed on an C18 analytical column ((2.1 mm × 100 mm, i.d., 1. 9 μm). All samples were eluted with water with 0.1% formic acid solution (mobile phase A) and 100% methanol (mobile phase B) at 25 °C at a low-rate of 0.3 mL/min. Conditions for mass spectral analysis in the positive ion mode included a capillary voltage of 4000 V, a nebulizing pressure of 2.8 × 10^5^ Pa, a drying gas flow of 10 L/min, and a temperature of 350 °C.

### RNA extraction, library construction and RNA-Seq

Total RNA of *P. massoniana* was extracted using RNAprep Pure Plant Kit (Polysaccharides & Polyphenolics-rich) (Tiangen, Beijing, China) following the manufacturer’s instructions. DNase I (Promega, Madison, USA) was used to digest extracted RNA to remove DNA contamination. The purity, concentration and integrity of RNA was measured according to methods of Nanodrop, Qubit 2.0, Aglient 2100. Purification of mRNA was carried out according to the method described in Dynabeads mRNA Purification Kit (Invitrogen, Carlsbad, USA). The cDNA libraries were constructed and sequenced using an illumina HiSeq2500 in Novogene Bioinformatics Technology Co., Ltd, Beijing, China.

### De novo assembly and functional annotation

The clean data were assembled into unigenes using Trinity software^54^. BlastX alignment between unigenes and NR、Swiss-Prot, GO, COG, KOG, KEGG were performed. Amino acids of unigenes were aligned to the Pfam database^55^ using HMMER^56^ to obtain annotation. Coding sequences of unigenes were predicted by TransDecoder.

### Differential expression analysis

Aligement between Reads and unigenes was performed using Bowtie^57^ and gene expression levels were estimated by RSEM. Unigene expression abundance was calculated using the FPKM method. Pearson’s Correlation Coefficient (r) was used to evaluate the index of biological repeat correlation^58^. Differential expression analysis between sample groups was performed using DESeq^59^. The *p*-values were adjusted by Benjamini and Hochberg’s approach to minimize false discovery rate. The differentially expressed genes (DEGs) were identified according to criteria: an adjusted *p*-value ≤ 0.05, a FDR < 0.01, a Fold Change ≥ 2.

### Functional annotation and GO enrichment analysis

We imported Nr results into Blast2GO program and get all the selected genes with annotated GO terms. WEGO software was used to classify the GO terms. GO enrichment was performed by topGO. We assigned the assembled sequences by KEGG pathway.

### Quantitative real-time polymerase chain reaction (qRT-PCR)

Primers were designed for 52 isoform sequences (Table S11). *actin1* gene was used as an internal control. ACT1 was amplified through the following primers as standard control: 5′ -CAGTGTCTGGATTGGAGGTTC-3′ as primer 1 and 5′ –TCTGTGGACGATGGAAGGAC-3′ as primer 2^60^. Reserve transcription of mRNA was done according to manufacturer’s instructions of M-MLV. qRT-PCR reactions were performered using SYBR Premix Ex Taq II (TaKaRa) on a Light Cycle 480 II system. The relative expression level was calculated by means of 2^–ΔΔCt^ method^61^.

## Supplementary Information

Supplementary Information 1.

Supplementary Information 2.

Supplementary Information 3.

Supplementary Information 4.

Supplementary Information 5.

Supplementary Information 6.

Supplementary Information 7.

Supplementary Information 8.

Supplementary Information 9.

Supplementary Information 10.

Supplementary Information 11.

Supplementary Information 12.

Supplementary Information 13.

Supplementary Information 14.

Supplementary Information 15.

Supplementary Information 16.

Supplementary Information 17.

Supplementary Information 18.

## References

[1] Evers JB (2011). Understanding shoot branching by modelling form and function. Trends Plant Sci..

[2] Domagalska MA (2011). Signal integration in the control of shoot branching. Nat. Rev. Mol. Cell Biol..

[3] Ongaro V (2008). Interactions between axillary branches of *Arabidopsis*. Mol. Plant.

[4] Beveridge CA (2000). Auxin inhibition of decapitation -induced branching is dependent on graft-transmissible signals regulated by genes Rms1 and Rms2. Plant Physiol..

[5] Booker J (2005). MAX1 encodes a cytochrome P450 family member that acts downstream of MAX3/4 to produce a carotenoid-derived branch- inhibiting hormone. Dev. Cell.

[6] Leyser O (2009). The control of shoot branching: An example of plant information processing. Plant Cell Environ..

[7] Thimann KV (1934). On the inhibition of bud development and other functions of growth substance in *Vicia faba*. Proc. R. Soc. Lond. B..

[8] Blakeslee JJ (2005). Auxin transport. Curr. Opin. Plant Biol..

[9] Snow R (2010). The young leaf as the inhibiting organ. New Phytol..

[10] Vieten A (2007). Molecular and cellular aspects of auxin- transport-mediated development. Trends Plant Sci..

[11] Pozo DJC (2002). AXR1-ECR1-dependent conjugation of RUB1 to the Arabidopsis cullin AtCUL1 is required for auxin response. Plant Cell.

[12] Umehara M (2008). Inhibition of shoot branching by new terpenoid plant hormones. Nature.

[13] Ruyter-Spira C (2013). The biology of strigolactones. Trends Plant Sci..

[14] Gomez-Roldan V (2008). Strigolactone inhibition of shoot branching. Nature.

[15] Abe S (2014). Carlactone is converted to carlactonoic acid by MAX1 in Arabidopsis and its methyl ester can directly interact with AtD14 in vitro. Proc. Natl. Acad. Sci. USA.

[16] Ferguson BJ (2009). Roles for auxin, cytokinin, and strigolactone in regulating shoot branching. Plant Physiol..

[17] Du Y (2017). UNBRANCHED3 regulates branching by modulating cytokinin biosynthesis and signaling in maize and rice. New Phytol..

[18] Ni J (2015). Gibberellin promotes shoot branching in the perennial woody plant *Jatropha curcas*. Plant Cell Physiol..

[19] Brandt B (2012). Reconstitution of abscisic acid activation of SLAC1 anion channel by CPK6 and OST1 kinases and branched ABI1 PP2C phosphatase action. Proc. Nati. Acad. Sci. USA.

[20] Robrecht D (2018). Branching gene expression during chrysanthemum axillary bud outgrowth regulated by strigolactone and auxin transport. Plant Growth Regul..

[21] Nie J (2018). The AP2/ERF transcription factor CmERF053 of chrysanthemum positively regulates shoot branching, lateral root, and drought tolerance. Plant Cell Rep..

[22] Guo D (2015). The WRKY transcription factor WRKY71/EXB1 controls shoot branching by transcriptionally regulating\r, RAX\r, genes in *Arabidopsis*. Plant Cell.

[23] Gao R (2018). SPL13 regulates shoot branching and flowering time in *Medicago sativa*. Plant Mol. Biol..

[24] Pan X (2016). ZmCCD7/ZpCCD7 encodes a carotenoid cleavage dioxygenase mediating shoot branching. Planta.

[25] Edelman SM (2018). Review of vegetative branching in the palms (*Arecaceae*). Bot. Rev..

[26] Lei C (2018). Effects of light on branch growth and death vary at different organization levels of branching units in *Sakhalin spruce*. Trees.

[27] Muhr M (2016). Knockdown of strigolactone biosynthesis genes in Populus affects BRANCHED1 expression and shoot architecture. New Phytol..

[28] Zheng K (2016). Characterization of DWARF14 genes in *Populus*. Sci. Rep..

[29] Wang J (2019). Transcriptome sequencing of active buds from *Populus deltoides* CL. and Populus × zhaiguanheibaiyang reveals phytohormones involved in branching. Genomics.

[30] Fan FH (2014). LTR-retrotransposon activation, IRAP marker development and its potential in genetic diversity assessment of masson pine (*Pinus massoniana*). Tree.

[31] Wang XZ (2015). Identification and genetic analysis of the pinewood nematode *Bursaphelenchus xylophilus* from *Pinus yunnanensis*. For. Pathol..

[32] Fan FH (2014). The Temporal transcriptomic response of *Pinus massoniana* seedlings to phosphorus deficiency. PLoS ONE.

[33] Zhao GW (2014). Roles of gibberellin and auxin in promoting seed germination and seedling vigor in *Pinus massoniana*. For. Sci..

[34] Metzger RJ (1999). Genetic control of branching morphogenesis. Science.

[35] Hayward A (2009). Interactions between auxin and strigolactone in shoot branching control. Plant Physiol..

[36] Jiang B (2012). Changes of endogenous hormones in lateral buds of chrysanthemum during their outgrowth. Russ. J. Plant Physiol..

[37] Crawford S (2010). Strigolactones enhance competition between shoot branches by dampening auxin transport. Development.

[38] Liang J (2010). Strigolactone regulation of shoot branching in chrysanthemum (*Dendranthema grandiflorum*). J. Exp. Bot..

[39] Yao R (2016). DWARF14 is a non-canonical hormone receptor for strigolactone. Nature.

[40] Zhou F (2013). D14–SCFD3-dependent degradation of D53 regulates strigolactone signalling. Nature.

[41] Bennett T (2006). The arabidopsis MAX pathway controls shoot branching by regulating auxin transport. Curr. Biol..

[42] Lo SF (2008). A novel class of Gibberellin 2-oxidases control semidwarfism, tillering, and root development in rice. Plant Cell.

[43] Geuns JM (2001). Apical dominance in Pssu-ipt-transformed tobacco. Phytochemistry.

[44] Bakshi M (2014). WRKY transcription factors: Jack of many trades in plants. Plant Signal. Behav..

[45] Guo D (2016). EXB1/WRKY71 transcription factor regulates both shoot branching and responses to abiotic stresses. Plant Signal. Behav..

[46] Journot-Catalino N (2006). The transcription factors WRKY11 and WRKY17 act as negative regulators of basal resistance in *Arabidopsis thaliana*. Plant Cell.

[47] Devaiah BN (2007). WRKY75 transcription factor is a modulator of phosphate acquisition and root development in Arabidopsis. Plant Physiol..

[48] Poza-Carrión C (2007). Role of TCP gene BRANCHED1 in the control of shoot branching in Arabidopsis. Plant Signal. Behav..

[49] Braun N (2012). The pea TCP transcription factor PsBRC1 acts downstream of strigolactones to control shoot branching. Plant Physiol..

[50] Naz AA (2013). Trifoliate encodes an MYB transcription factor that modulates leaf and shoot architecture in tomato. Proc. Nati. Acad. Sci. USA.

[51] Lee DK (2009). LATERAL ORGAN FUSION1 and LATERAL ORGAN FUSION2 function in lateral organ separation and axillary meristem formation in *Arabidopsis*. Development.

[52] Mehrnia M (2013). An AP2/ERF transcription factor highly expressed in Proliferating cells, affects shoot architecture in *Arabidopsis*. Plant Physiol..

[53] Martín-Trillo M (2010). TCP genes: A family snapshot ten years later. Trends Plant Sci..

[54] Grabherr MG (2011). Full length transcriptome assembly from RNA Seq data without a reference genome. Nat. Biotechnol..

[55] Finn RD (2013). Pfam: The protein families database. Nucleic Acids Res..

[56] Eddy SR (1998). Profile hidden markov models. Bioinformatics.

[57] Langmead B (2009). Ultrafast and memory-efficient alignment of short DNA sequences to the human genome. Genome Biol..

[58] Schulze SK (2012). SERE: Single-parameter quality control and sample comparison for RNA-Seq. BMC Genom..

[59] Anders S (2010). Differential expression analysis for sequence count data. Genome Biol..

[60] Chen H (2015). Reference genes selection for quantitative gene expression studies in *Pinus massoniana* L. Trees.

[61] Livak KJ (2001). Analysis of relative gene expression data using real-time quantitative PCR and the 2^−ΔΔCT^ method. Methods.

