# Supplementary material for: Molecular mechanism of lateral bud differentiation of Pinus massoniana based on high-throughput sequencing
Source: Sci Rep. 2021 Apr 27;11:9033. doi: 10.1038/s41598-021-87787-7 (PMC8079368; doi:10.1038/s41598-021-87787-7)
Supplement: Supplementary file 1 — Supplementary Information 1. [file 41598_2021_87787_MOESM1_ESM.docx]

**Molecular mechanism of lateral bud differentiation of *Pinus massoniana* based on high-throughput sequencing**

Hu Chen^1,2,3,4^, Jianhui Tan^1,3^, Xingxing Liang^1^, Shengsen Tang^1,2^, Jie Jia^1,3,4^, Zhangqi Yang ^1,2,3,4※^

1.Guangxi Forestry Research Institute of Science, Nanning 530002, PR China

2.Guangxi Key Laboratory of Superior Timber Trees Resource Cultivation, Nanning 530002, PR China

3. Engineering research center of Masson Pine of state forestry administration, Nanning 530002, PR China

4. Engineering research center of Masson Pine of Guangxi, Nanning 530002, PR China

※Corresponding authors. Fax: +86 +0771+2319901

E-mail address: yangzhangqi@163.com

**Supplementary information accompanies this paper at**

**Figure S1.** Distribution of unigene lengths

The X-axis indicated the different length intervals of unigenes; the Y-axis indicated the number of unigenes in a certain length interval

**Figure S2.** GO、KEGG 、COG classification of unigenes

The results are summarized in three main GO categories: Biological process, Cellular Component and Molecular Function. KEGG enrichments of the annotated DEGs across two comparisons. The left Y-axis indicates the KEGG pathway. The X-axis indicates the Rich factor.COG function classification of consensus sequence.

**Figure S3.** Scatter plot of gene expression of two samples

Each point representd a gene, and the X-axis and Y-axis were the logarithm of FPKM+1. The more the point deviates from the diagonal, the greater the difference in the expression level of corresponding genes between two samples. In addition, the more points deviating from the diagonal, the lower the correlation between expression levels of two samples, and the greater the difference in expression levels; and vice versa.

**Figure S4.** Annotations of all unigenes using MapMAN software

**Figure S5.** Differential expression of transcription factor-related genes

The pathway was generated using MapMAN software (Version 3. 6.0RC1). Totally 901 genes were involved in different transcription factor pathway. Each square means a gene. Blue color means up-regulation and red color means down-regulation. a: Differential expression data from sample L v T, b: Differential expression data from sample K v T.

**Figure S6.** Differential expression of secondary metabolic related genes

The pathway was generated using MapMAN software (Version 3. 6.0RC1). Totally 701 genes were involved in different pathway. Each square means a gene. Blue color means up-regulation and red color means down-regulation. a: Differential expression data from sample L v T, b. Differential expression data from sample K v T.

**Table S1.** Fluorescent quantitative genes and primer sequence information

**Table S2.** Evaluation of sequencing data

Samples: sample name; Read Number: total number of pair-end Reads in clean data; Base Number: total number of bases in clean data; GC Content: Clean Data GC content, ie, G and C bases in clean data percentage of total bases; %≥Q30: Percentage of bases with a clean data mass value greater than or equal to 30.

**Table S3.** Statistics table of assembly result

**Table S4.** Statistics of unigene annotations

Gene annotations were selected according to E-value≤10^-5^ in BLAST and E-value≤10^-10^ in HMMER.

**Table S5.** KEGG_enrichment

**Table S6.** Numbers of differential expression genes

DEG Set: name of differential expression gene set; All DEG: number of differentially expressed genes; up-regulated: number of up-regulated genes; down-regulated: number of down-regulated genes.

**Table S7.** DEGs information obtained by veen analysis using MapManExport software

**Table S8.** Statistics of annotated differential expression gene

**Table S9.** Enrichment of differentially expressed genes of KEGG annotated hormone signaling pathway

**Table S10.** Differentially expressed genes in the metabolic pathway of each transcription factor

**Table S11.** Gene information of secondary metabolic genes in metabolic pathways of various transcription factors
