# Supplementary figures and images for: Molecular mechanism of lateral bud differentiation of Pinus massoniana based on high-throughput sequencing
Source: Sci Rep. 2021 Apr 27;11:9033. doi: 10.1038/s41598-021-87787-7 (PMC8079368; doi:10.1038/s41598-021-87787-7)

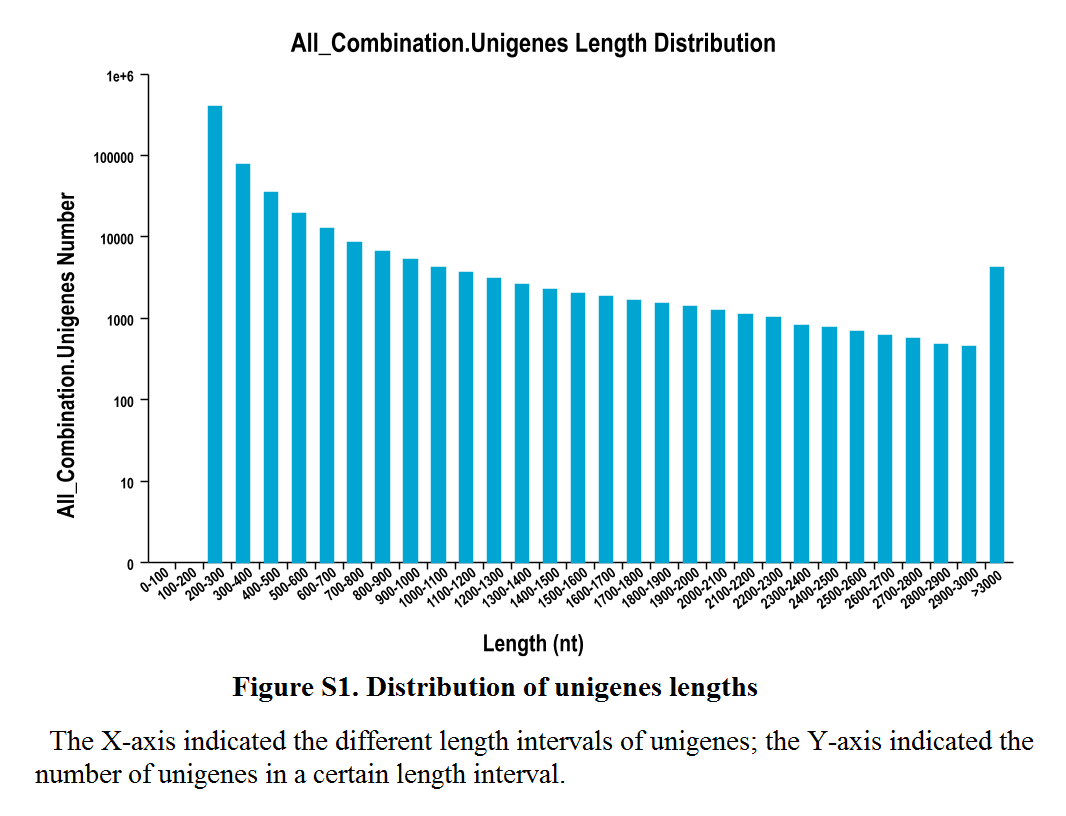

Supplement: Supplementary file 2 — Supplementary Information 2. [file 41598_2021_87787_MOESM2_ESM.png]

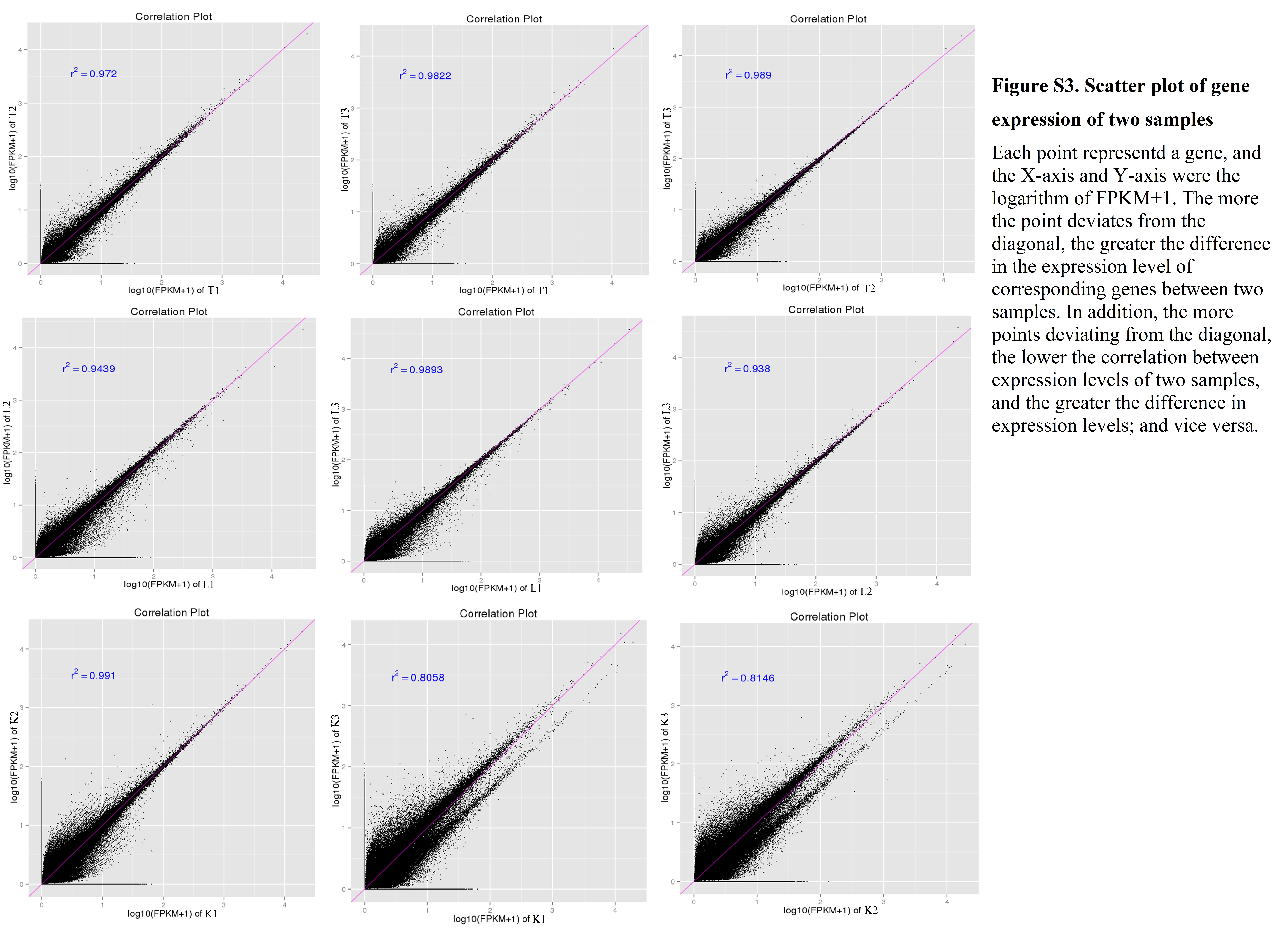

Supplement: Supplementary file 4 — Supplementary Information 4. [file 41598_2021_87787_MOESM4_ESM.jpg]

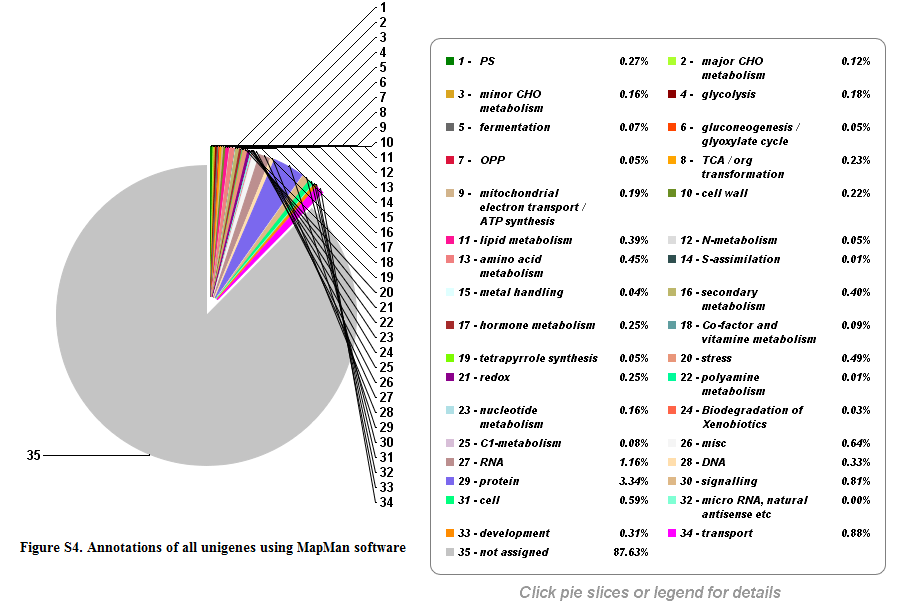

Supplement: Supplementary file 5 — Supplementary Information 5. [file 41598_2021_87787_MOESM5_ESM.png]

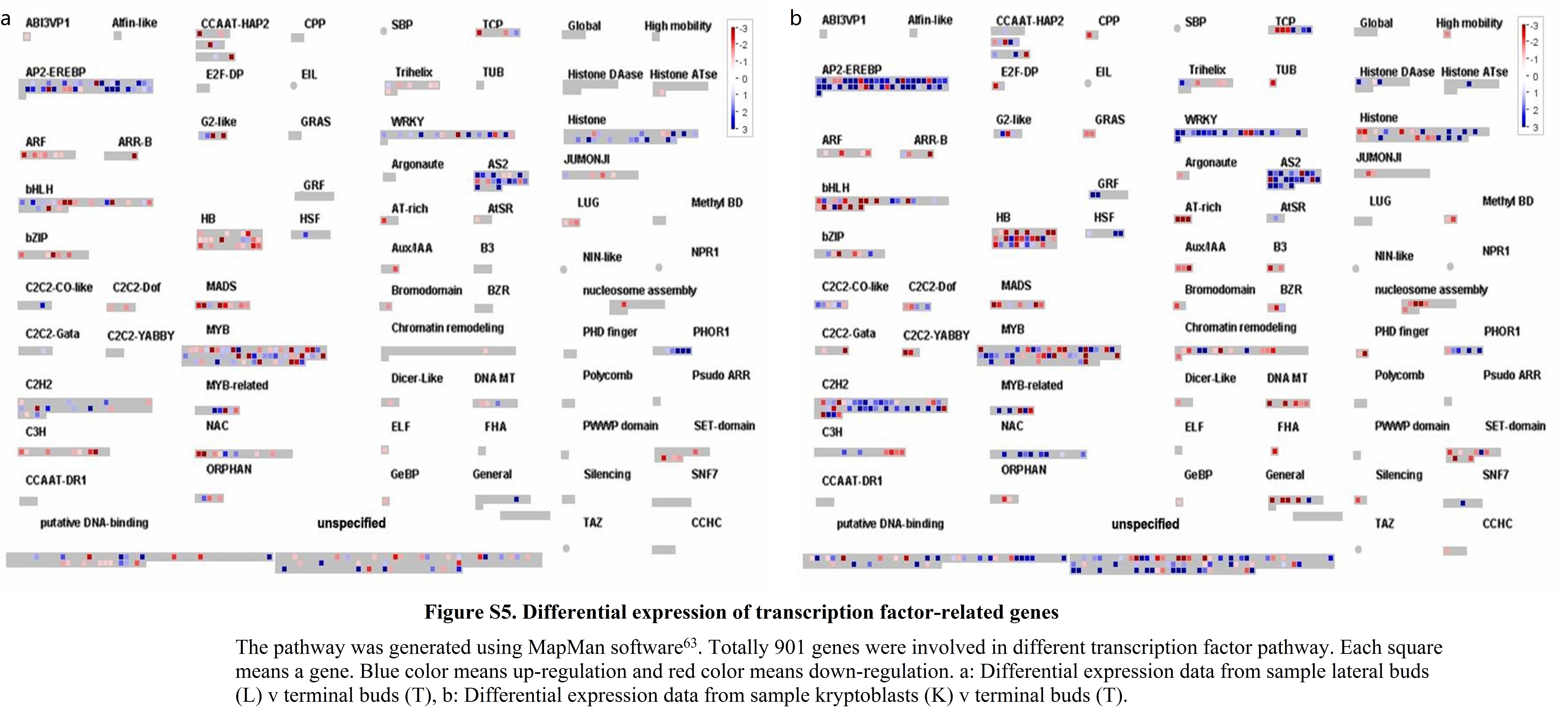

Supplement: Supplementary file 6 — Supplementary Information 6. [file 41598_2021_87787_MOESM6_ESM.jpg]

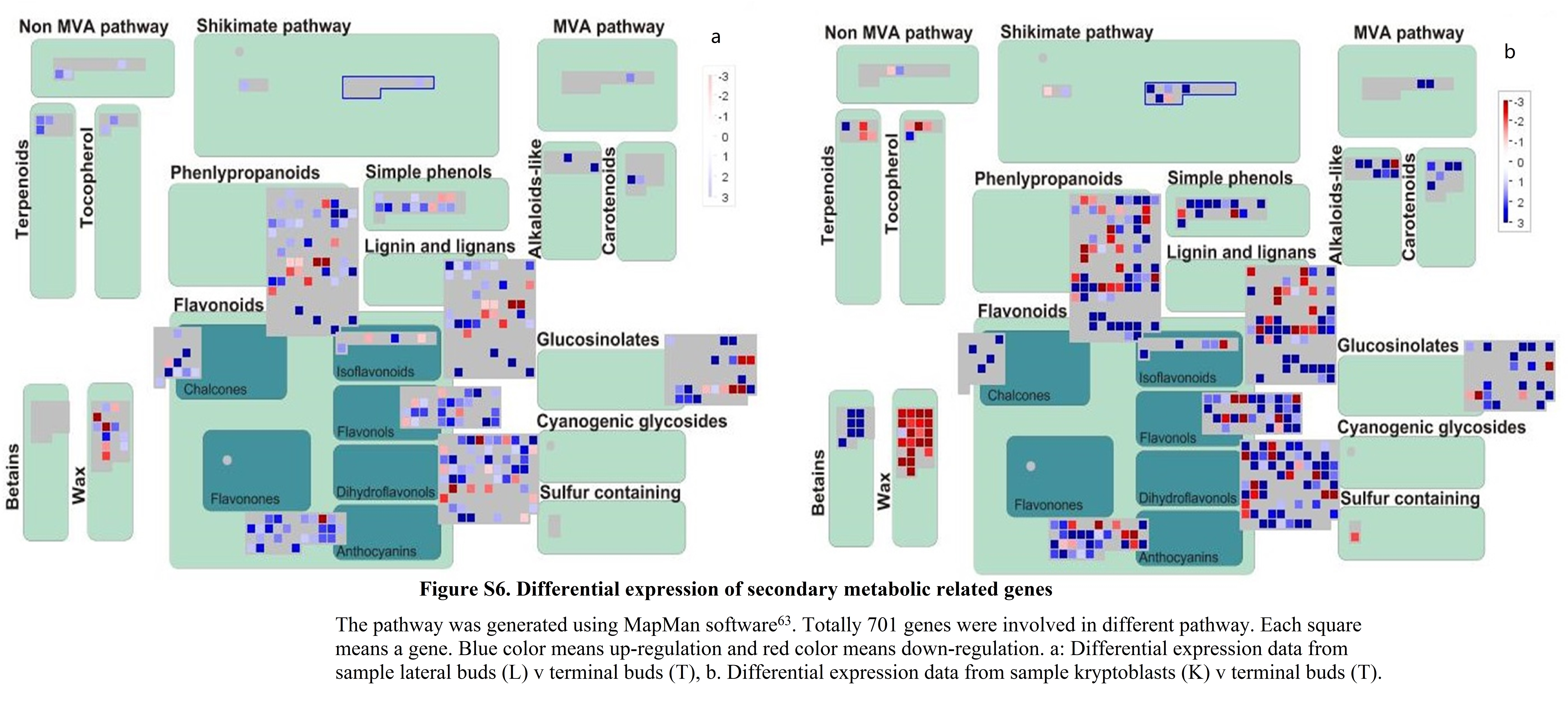

Supplement: Supplementary file 7 — Supplementary Information 7. [file 41598_2021_87787_MOESM7_ESM.jpg]
